# The Effect of immunotherapy on oligometastatic non-small cell lung cancer patients by sites of metastasis

**DOI:** 10.3389/fimmu.2022.1039157

**Published:** 2022-11-14

**Authors:** Jia-Chun Ma, Jing-Xin Zhang, Fei Wang, Jinming Yu, Dawei Chen

**Affiliations:** ^1^ Lung Cancer Center, West China Hospital, Sichuan University, Chengdu, China; ^2^ Department of Radiation Oncology and Shandong Provincial Key Laboratory of Radiation Oncology, Shandong Cancer Hospital and Institute, Shandong First Medical University and Shandong Academy of Medical Sciences, Jinan, China; ^3^ Research Unit of Radiation Oncology, Chinese Academy of Medical Sciences, Jinan, China

**Keywords:** non-small cell lung cancer, immunotherapy, oligometastases, overall survival, metastatic sites

## Abstract

**Introduction:**

The efficacy of immunotherapy for treatment of patients with oligometastatic non-small cell lung cancer (NSCLC) at different metastatic sites remains controversial. We investigated the effect of different metastatic sites on immunotherapy for oligometastatic NSCLC following local treatment (LT).

**Methods:**

We retrospectively analyzed patients with oligometastatic NSCLC from the latest 2018 registry on the SEER Stat software (8.3.9. Version) and a Chinese single-center cohort. The effects of immunotherapy on OS (overall survival) and CSS (cancer specific survival) were estimated for patients with different metastatic sites.

**Results:**

A total of 483 patients in the SEER-18 database and 344 patients in the **single-center** cohort were included. Immunotherapy was significantly correlated with improved OS (SEER: Hazard ratio 0.754, 95% CI 0.609–0.932; P=0.044; China: Hazard ratio 0.697, 95% CI 0.542–0.896; P=0.005) and CSS (SEER: Hazard ratio 0.743, 95% CI 0.596–0.928; P=0.009; China: Hazard ratio 0.725, 95% CI 0.556–0.945; P=0.018). Subgroup analysis showed that OS was improved after immunotherapy in the BRM (SEER: Hazard ratio 0.565, 95% CI 0.385–0.829; P=0.004; China: Hazard ratio 0.536, 95% CI 0.312–0.920; P=0.024) and MOM (SEER: Hazard ratio 0.524, 95% CI 0.290–0.947; P=0.032; China: Hazard ratio 0.469, 95% CI 0.235–0.937; P=0.032) subgroups, but not in the BOM (SEER: P=0.334; China: P=0.441), LIM (SEER: P=0.301; China: P=0.357), or OTM (SEER: P=0.868; China: P=0.489) subgroups.

**Conclusions:**

This study showed that immunotherapy conferred survival benefits on patients with oligometastatic NSCLC. Our subgroup analysis suggested that patients with oligometastatic NSCLC in the brain or multiple organs may particularly benefit from aggressive front-line therapies.

## Introduction

Non-small cell lung cancer accounts for 85% of lung cancer and is the leading cause of cancer-related mortality (1). Non-small cell lung cancer is the primary cause of cancer-related death in the world mainly because patients with lung cancer often develop advanced metastases (2). Approximately 25–50% of patients with NSCLC exhibit an oligometastatic status (3). The concept of “oligometastasis” builds on two models of cancer progression: the Halsted model of continuous progression and a systemic model that hypothesizes that disease is a manifestation of extensive systemic clinical involvement (4). Patients with oligometastatic NSCLC may achieve long-term disease control and may even be cured. In retrospective studies, survival in patients with oligometastatic NSCLC had better overall survival (OS) than patients with a large number of metastases (3, 5). Therefore, it is important to investigate selection of treatment for these patients with limited metastatic NSCLC.

A number of previous studies (6–8) on oligometastatic NSCLC have shown that use of local treatment (LT) at all metastatic sites resulted in longer OS and progression-free survival (PFS) than those following palliative treatment. Immunotherapy has become the standard treatment for patients with metastatic NSCLC (9). The KEYNOTE-189 (10) and KEYNOTE-407 (11) studies have shown that pembrolizumab in combination with specific chemotherapy improved OS and PFS in patients with untreated metastatic NSCLC, regardless of PD-L1 expression. Therefore, use of LT combined with immunotherapy for treatment of oligometastatic NSCLC is an area of active investigation.

Joshua M et al (12) reported in a phase II clinical study that pembrolizumab improved PFS in patients with oligometastatic NSCLC after LT. Although this study showed that pembrolizumab could improve the prognosis of patients with oligometastatic NSCLC, this study did not consider the effect of factors such as the location and the number of organs affected by oligometastases on prognosis, and there are no phase III clinical prospective trials focused on oligometastatic NSCLC. Several previous studies have confirmed that the prognosis of patients with oligometastatic NSCLC was closely related to the site and number of metastatic organs (3, 13, 14). Ashworth AB et al (14) reported that prognosis in patients with oligometastatic NSCLC was associated with brain metastasis but did not address the relationship between prognosis and other metastatic sites.

No study has elucidated the effects of immunotherapy in patients with oligometastatic NSCLC at different metastatic sites. Therefore, our goal was to investigate the effect of different metastatic sites on immunotherapy for oligometastatic NSCLC following LT.

## Methods

### Patient cohort

**Figure 1:**
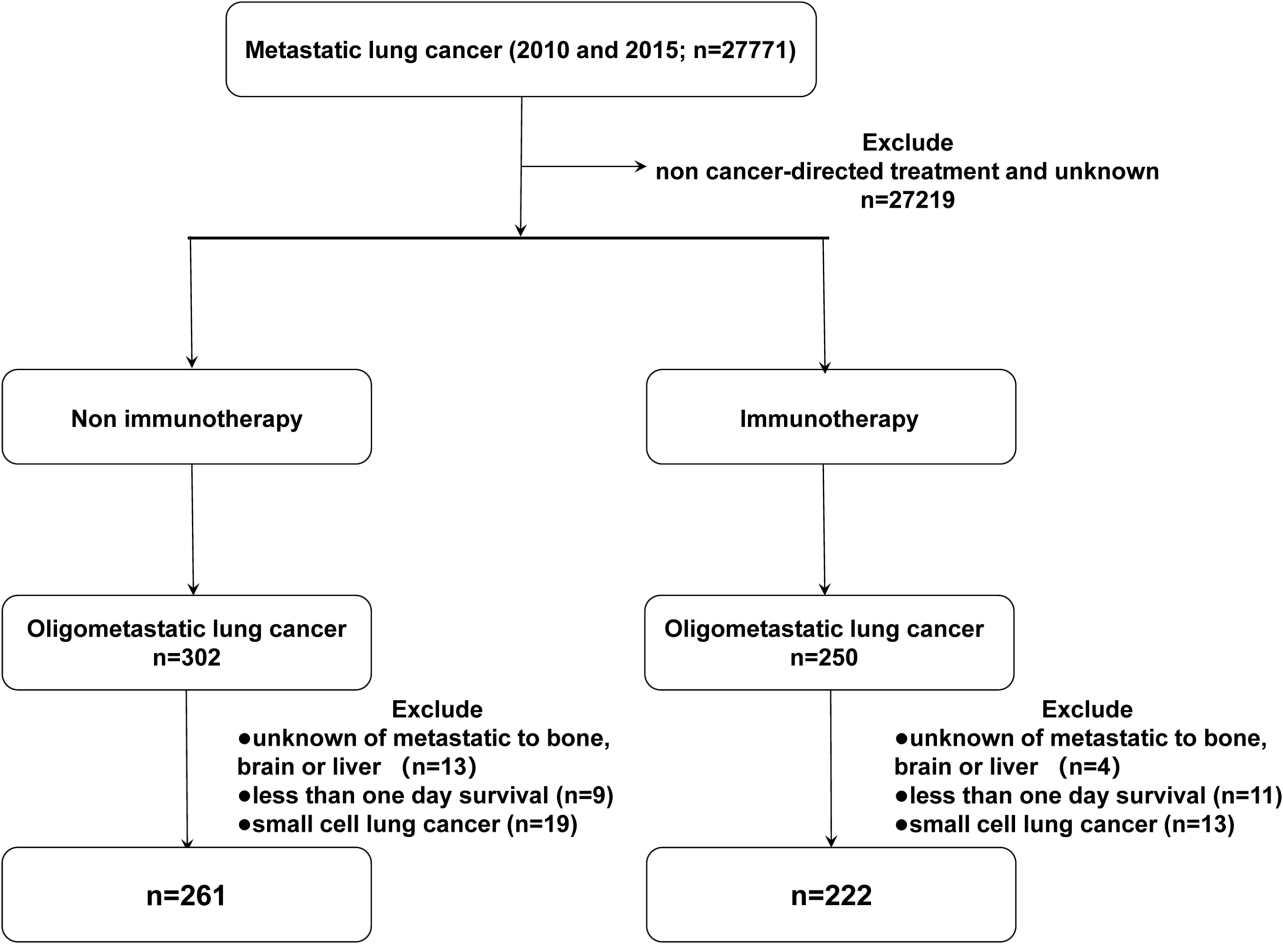
Flow diagram of the patient selection process from the SEER Database.

Patient data were collected from the incidence-SEER 18 registries from the National Cancer Institute SEER Stat software (Version 8.3.9), with additional treatment fields added. According to the third edition of the International Classification of Diseases for Oncology (ICD-O-3) and the AJCC 7th TNM staging of NSCLC, we screened patients who were first primarily diagnosed with malignant tumors of the lung and bronchus with M1b stage in 2010 and 2015. We selected patients with oligometastatic NSCLC based on the time of approval of immunotherapy as the primary treatment (2015), with patients diagnosed in 2010 as a comparison. The inclusion criteria were as follows: 1) patients with metastatic non-small cell lung cancer treated with cancer-directed treatment; 2) no younger than 20 years old. The exclusion criteria were: 1) more than one primary tumor; 2) incomplete data on metastases of the bone, liver, and brain at diagnosis; 3) incomplete treatment and follow-up information; 4) 0 days of survival. The following variables were selected: ID number, age, sex, year of diagnosis, race (white, black, or other), marital status, site recode ICD-0-3, affected side, tissue grade, histology, T stage (AJCC 7th), N stage (AJCC 7th), metastases of the bone, liver, and brain at diagnosis, radiation (radiation or other), chemotherapy (yes or no), surgery information, survival months, COD to site rec KM, vital status record. Patients were divided into five subgroups: BOM (bone metastasis), BRM (brain metastasis), LIM (liver metastasis), MOM (multiple organ metastasis), and OTM (other metastasis). The detailed inclusion and exclusion criteria are summarized in **Figure 1**.

An independent cohort of patients with oligometastatic NSCLC from 2016 to 2019 from Shandong Cancer Hospital in China was used for external validation. These patients met the same inclusion and exclusion criteria as those from the SEER Database.

### Statistical analysis

The Pearson chi-squared test was used to compare demographics, pathology, and site of metastasis in patients with or without immunotherapy. We chose 36 months as the cutoff value. Overall survival and CSS were analyzed using the Kaplan–Meier (KM) method *via* the log-rank test. Univariate and multivariate analyses was performed using the Cox proportional hazards risk model. Variables that were significantly associated with survival in the univariate analysis were included in the multivariate Cox analysis. All tests were two-sided tests, and P <0.05 was considered statistically significant. SPSS 26 (IBM, Armonk, USA) and R 4.1.2 (R Foundation for Statistical Computing, Vienna, Austria) were used for analysis.

## Results

### Patient characteristics

A total of 483 patients in the SEER cohort and 344 patients in the single-center cohort who met the eligibility criteria were included in this study. The baseline characteristics of each cohort are listed in **Tables 1** and **2**. In the SEER cohort, at a median follow-up of 24.8 months (range, 1-107 months), 370 patients died, among whom 339 died of lung cancer. And in the single-center cohort, 245 deaths, including 219 cases dying from NSCLC with a median follow-up of 20.3 months (range, 3-53 months).

**Table 1:** Baseline characteristics of all patients in the SEER and the single-center cohorts.

Characteristics	SEER CohortN=483(%)	Single-Center CohortN=344(%)	*P*
**Age**			
<65	262 (54.2%)	120 (34.9%)	<0.001
‗65	221 (45.8%)	224 (65.1%)	
**Sex**			
Male	275 (56.9%)	198 (57.6%)	0.859
Female	208 (43.1%)	146 (42.4%)	
**Origin**			
Left	205 (42.4%)	136 (39.5%)	0.402
Right	278 (57.6%)	208 (60.5%)	
**Histology**			
Squamous cell carcinoma	146 (30.2%)	119 (34.6%)	0.185
Others	337 (69.8%)	225 (65.4%)	
**T stage**			
T1	119 (24.6%)	44 (12.8%)	<0.001
T2	85 (17.6%)	113 (32.8%)	
T3	168 (34.8%)	143 (41.6%)	
T4	108 (22.4%)	38 (11.0%)	
Unknown	3 (0.6%)	6 (1.7%)	
**N stage**			
N0	200 (41.4%)	161 (46.8%)	0.201
N1	64 (13.3%)	41 (11.9%)	
N2	147 (30.4%)	91 (26.5%)	
N3	56 (11.6%)	46 (13.4%)	
Unknown	16 (3.3%)	5 (1.5%)	
**Radiation status**			
No	217 (44.9%)	159 (46.2%)	0.713
Yes	266 (55.1%)	185 (53.8%)	
**Immunotherapy**			
No	261 (54.0%)	163 (47.4%)	0.059
Yes	222 (46.0%)	181 (52.6%)	
**Bone metastasis**			
No	323 (66.9%)	218 (63.4%)	0.297
Yes	160 (33.1%)	126 (36.6%)	
**Brain metastasis**			
No	283 (58.6%)	216 (62.8%)	0.224
Yes	200 (41.4%)	128 (37.2%)	
**Liver metastasis**			
No	400 (82.8%)	268 (77.9%)	0.094
Yes	83 (17.2%)	76 (22.1%)	
**Metastasis site**			
BOM	103 (21.3%)	79 (23.0%)	0.274
BRM	160 (33.1%)	82 (23.8%)	
LIM	45 (9.3%)	30 (8.7%)	
MOM	59 (12.2%)	67 (19.5%)	
OTM	116 (24.0%)	86 (25.0%)	

BOM, bone metastases only; BRM, brain metastases only; LIM, liver metastases only; MOM, multiple organ metastases; OTM, other metastases.

*P value is significant

**Table 2:** Characteristics of all patients according to receipt of immunotherapy in each cohort.

Variable	SEER Cohort			Single-Center Cohort		
	No immunotherapyN=261 (%)	ImmunotherapyN=222 (%)	P	No immunotherapyN=163 (%)	ImmunotherapyN=181 (%)	P
**Age**						
<65	146 (55.9%)	116 (52.3%)	0.418	56 (34.4%)	64 (35.4%)	0.845
‗65	115 (44.1%)	106 (47.7%)		107 (65.6%)	117 (64.6%)	
**Sex**						
Male	152 (58.2%)	123 (55.4%)	0.531	98 (60.1%)	100 (55.2%)	0.361
Female	109 (41.8%)	99 (44.6%)		65 (39.9%)	81 (44.8%)	
**Origin**						
Left	111 (42.5%)	94 (42.3%)	0.967	68 (41.7%)	68 (37.6%)	0.432
Right	150 (57.5%)	128 (57.7%)		95 (58.3%)	113 (62.4%)	
**Histology**						
Squamous cell carcinoma	89 (34.1%)	57 (25.7%)	0.056	60 (36.8%)	59 (32.6%)	0.412
Others	172 (65.9%)	165 (74.3%)		103 (63.2%)	122 (67.4%)	
**T stage**						
T1	69 (26.4%)	50 (22.5%)	0.086	28 (17.2%)	16 (8.8%)	0.110
T2	45 (17.2%)	40 (18.0%)		52 (31.9%)	61 (33.7%)	
T3	95 (36.4%)	73 (32.9%)		65 (39.9%)	78 (43.1%)	
T4	49 (18.8%)	59 (26.6%)		14 (8.6%)	24 (13.3%)	
Unknown	3 (1.1%)	0 (0.0%)		4 (2.5%)	2 (1.1%)	
**N stage**						
N0	114 (43.7%)	86 (38.7%)	0.483	82 (50.3%)	79 (43.6%)	0.666
N1	34 (13.0%)	30 (13.5%)		18 (11.0%)	23 (12.7%)	
N2	81 (31.0%)	66 (29.7%)		43 (26.4%)	48 (26.5%)	
N3	25 (9.6%)	31 (14.0%)		18 (11.0%)	28 (15.5%)	
Unknown	7 (2.7%)	9 (4.1%)		2 (1.2%)	3 (1.7%)	
**Radiation status**						
No	117 (44.8%)	100 (45.0%)	0.962	69 (42.3%)	90 (49.7%)	0.206
Yes	144 (55.2%)	122 (55.0%)		94 (57.7%)	91 (50.3%)	
**Bone metastasis**						
No	182 (69.7%)	141 (63.5%)	0.148	108 (66.3%)	110 (60.8%)	0.292
Yes	79 (30.3%)	81 (36.5%)		55 (33.7%)	71 (39.2%)	
**Brain metastasis**						
No	153 (58.6%)	130 (58.6%)	0.989	109 (66.9%)	107 (59.1%)	0.169
Yes	108 (41.4%)	92 (41.4%)		54 (33.1%)	74 (40.9%)	
**Liver metastasis**						
No	223 (85.4%)	185 (83.3%)	0.524	34 (20.9%)	42 (23.2%)	0.601
Yes	38 (14.6%)	37 (16.7%)		129 (79.1%)	139 (76.8%)	
**Metastasis site**						
BOM	55 (21.1%)	55 (21.6%)	0.225	40 (24.5%)	39 (21.5%)	0.563
BRM	92 (35.2%)	68 (30.6%)		41 (25.2%)	41 (22.7%)	
LIM	27 (10.3%)	18 (8.1%)		13 (8.0%)	17 (9.4%)	
MOM	24 (9.6%)	35 (15.8%)		26 (16.0%)	41 (22.7%)	
OTM	63 (23.8%)	53 (23.9%)		43 (26.4%)	43 (23.8%)	

BOM, bone metastases only; BRM, brain metastases only; LIM, liver metastases only; MOM, multiple organ metastases; OTM, other metastases.

*p value is significant

In the SEER cohort, 54.2% were aged <65. The majority of patients were men (56.9%). The distribution of the metastasis sites was 21.3%, 33.1%, 9.3%, 12.2%, and 24.0% for BOM, BRM, LIM, MOM, and OTM, respectively (**Table 1**). Moreover, this cohort enrolled 222 (46.0%) patients who received immunotherapy, 275 (57.0%) male patients, and 285 (59.0%) patients with adenocarcinoma histology. The different metastasis site subgroups were evenly distributed between the no immunotherapy and immunotherapy groups (**Table 2**). None of the factors, including age, sex, origin, histology, T stage, N stage, radiation status, chemotherapy, bone metastasis, brain metastasis, and liver metastasis differed significantly between the two groups (**Table 2**).

In the validation cohort, the metastasis sites included BOM (23.0%), BRM (23.8%), LIM (8.7%), MOM (19.5%), and OTM (25.0%) (**Table 2**). In this cohort, no factors, including metastasis-site subgroups, age, sex, origin, histology, T stage, or N stage correlated with immunotherapy.

### Immunotherapy and survival outcomes

**Figure 2:**
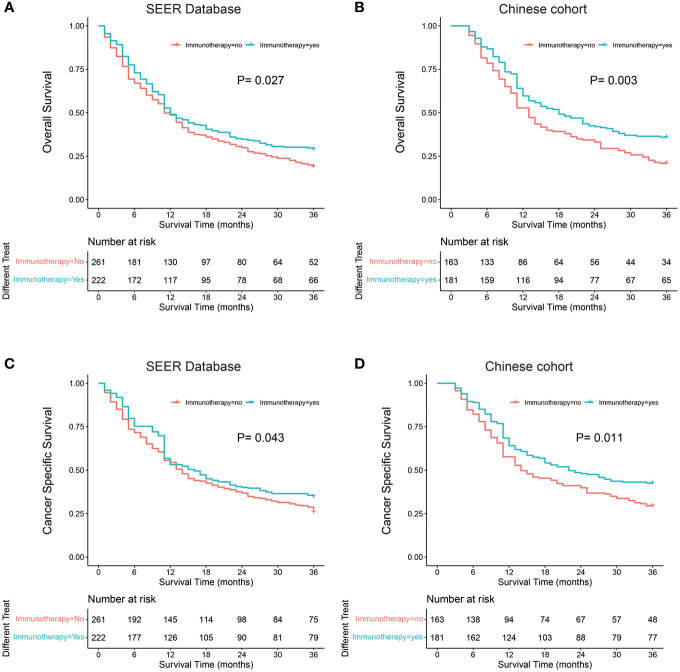
Kaplan-Meier probability plots of Overall Survival and Cancer Specific Survival between the Immunotherapy group and the No immunotherapy group: SEER Database **(A, C)** and Single-Center cohort **(B, D)**.

Kaplan–Meier probability plots showed improved OS and CSS after immunotherapy in both cohorts (**Figure 2**). We performed univariate and multivariate cox proportional hazards analyses to determine whether any clinical or pathological features were associated with OS and CSS (**Table 3**). In the univariate analysis, we found that immunotherapy was associated with better OS (SEER: Hazard ratio 0.797, 95% CI 0.649–0.980; P=0.027; China: Hazard ratio 0.693, 95% CI 0.539–0.890; P=0.004) and CSS (SEER: Hazard ratio 0.796, 95% CI 0.642–0.987; P=0.043; China: Hazard ratio 0.715, 95% CI 0.548–0.932; P=0.013). Moreover, we showed that sex, site, T stage, N stage, chemotherapy, and liver metastasis were significantly associated with clinical outcome in the univariate analysis. These factors were adjusted to evaluate the effect of immunotherapy. In the multivariate analysis, we found that immunotherapy was significantly correlated with improved OS (SEER: Hazard ratio 0.754, 95% CI 0.609–0.932; P=0.044; China: Hazard ratio 0.697, 95% CI 0.542–0.896; P=0.005) and CSS (SEER: Hazard ratio 0.743, 95% CI 0.596–0.928; P=0.009; China: Hazard ratio 0.725, 95% CI 0.556–0.945; P=0.018) (**Table 3**). Male patients had significantly worse OS (SEER: Hazard ratio 1.311, 95% CI 1.057–1.626; P=0.014; China: Hazard ratio 1.599, 95% CI 1.225–2.088; P=0.001) and CSS (SEER: Hazard ratio 1.282, 95% CI 1.020–1.610; P=0.033; China: Hazard ratio 1.502, 95% CI 1.143–1.976; P=0.004) than female patients in the multivariate analyses. Patients with lesions located in the main bronchus had a worse prognosis than those with lesions located at other sites. Patients with higher T and N stage showed better prognosis. Liver metastasis was associated with poor prognosis for the SEER cohort but not for the single-center cohort.

**Table 3:** Univariate and multivariate analyses of OS and CSS for different characteristics of oligometastatic NSCLC in each cohort.

	SEER Cohort							Single-Center Cohort							
Characteristics	Univariate analysis (OS)		Multivariate analysis (OS)					Univariate analysis (OS)						Multivariate analysis (OS)	
	HR (95% CI)	P	HR (95% CI)		P			HR (95% CI)		P			HR (95% CI)	P	
**Sex**															
Female	1.353 (1.098-1.666)	0.004*		1.311 (1.057-1.626)	0.014*		1.603 (1.237-2.077)			<0.001*		1.599 (1.225-2.088)		0.001*	
Male															
**T stage**		0.002*			0.013*					0.016*				0.126	
T1	Reference			Reference			Reference					Reference			
T2	0.878 (0.632-1.219)	0.437		0.850 (0.602-1.202)	0.359		0.968 (0.640-1.465)			0.878		0.911 (0.599-1.385)		0.838	
T3	1.120 (0.854-1.469)	0.414		1.234 (0.933-1.632)	0.140		1.041 (0.697-1.555)			0.844		0.989 (0.656-1.490)		0.827	
T4	1.608 (1.199-2.158)	0.002		1.475 (1.080-2.015)	0.015		1.775 (1.113-2.830)			0.016		1.501 (0.929-2.425)		0.076	
Unknown	1.693(0.535-5.361)	0.371		0.801(0.247-2.597)	0.711		2.006(0.706-5.698)			0.191		1.377(0.473-4.011)		0.558	
**N stage**		0.001*			<0.001*		*			0.015*				0.014*	
N0	Reference			Reference			Reference					Reference			
N1	1.347(0.977-1.857)	0.069		1.692(1.211-2.363)	0.002		1.222(0.815-1.832)			0.331		1.333(0.887-2.004)		0.166	
N2	1.389(1.084-1.779)	0.009		1.570(1.207-2.043)	0.001		0.979(0.705-1.360)			0.900		1.032(0.742-1.435)		0.853	
N3	1.944(1.412-2.676)	<0.001		2.062(1.471-2.890)	<0.001		1.714(1.221-2.406)			0.002		1.780(1.266-2.501)		0.001	
Unknown	1.255(0.711-2.216)	0.434		1.572(0.874-2.830)	0.131		1.834(0.748-4.501)			0.185		1.902(0.775-4.668)		0.161	
**Immunotherapy**															
No	0.797 (0.649-0.980)	0.031*	0.754 (0.609-0.932)		0.044*				0.693 (0.539-0.890)	0.004*		0.697 (0.542-0.896)		0.005*	
Yes									
	**Univariate analysis (CSS)**		**Multivariate analysis (CSS)**						**Univariate analysis (CSS)**			**Multivariate analysis (CSS)**			
	HR (95% CI)	P	HR (95% CI)		P				HR (95% CI)	P		HR (95% CI)		P	
**Sex**															
Female	1.363 (1.094-1.697)	0.006*	1.282 (1.020-1.610)			0.033*			1.519 (1.156-1.998)		0.003*	1.502 (1.143-1.976)			0.004*
Male															
**T stage**		0.005*				0.030*					0.024*				0.170*
T1	Reference		Reference						Reference			Reference			
T2	0.923(0.655-1.301)	0.647	0.884(0.618-1.264)			0.500			0.888(0.570-1.385)		0.601	0.825(0.526-1.294)			0.403
T3	1.154(0.867-1.537)	0.326	1.228(0.917-1.645)			0.168			1.080(0.705-1.654)		0.723	0.933(0.644-1.531)			0.974
T4	1.638(1.201-2.235)	0.002	1.414(1.019-1.962)			0.038			1.747(1.068-2.857)		0.026	1.438(0.867-2.386)			0.160
Unknown	0.636(0.088-4.581)	0.654	0.281(0.038-2.054)			0.221			1.405(0.427-4.626)		0.576	0.992(0.295-3.341)			0.990
**N stage**		0.001*				<0.001*					0.015*				0.014*
N0	Reference		Reference						Reference						
N1	1.466(1.049-2.049)	0.025	1.825(1.289-2.583)			0.001			1.397(0.921-2.119)		0.116	1.496(0.984-2.274)			0.060
N2	1.539(1.188-1.995)	0.001	1.747(1.329-2.297)			<0.001			1.046(0.740-1.477)		0.800	1.097(0.775-1.552)			0.601
N3	1.905(1.347-2.693)	<0.001	1.981(1.376-2.851)			<0.001			1.600(1.111-2.304)		0.012	1.637(1.136-2.359)			0.008
Unknown	1.342(0.741-2.429)	0.332	1.649(0.894-3.043)			0.110			2.254(0.916-5.550)		0.077	2.329(0.945-5.738)			0.066
**Immunotherapy**															
No	0.796 (0.642-0.987)	0.043*		0.743 (0.596-0.928)		0.009*			0.715 (0.548-0.932)		0.013*	0.725 (0.556-0.945)			0.018*
Yes													

*p value is significant.

### Subgroup analysis and validation

**Figure 3:**
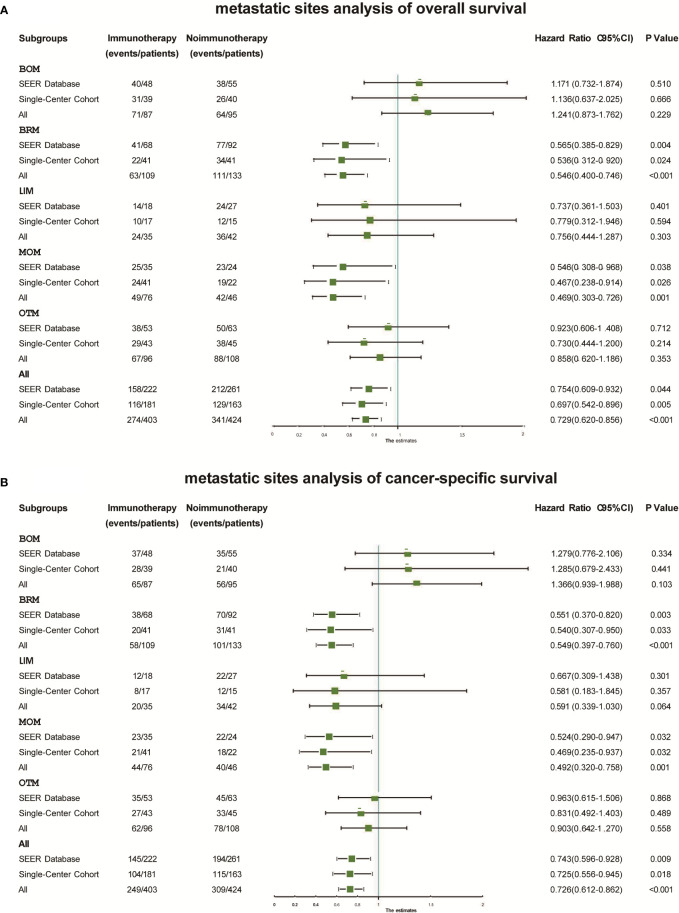
Forest plots displaying the relationship between immunotherapy and overall survival **(A)** and cancer-specific survival **(B)** within different subgroups in each cohort. BOM, bone metastases only; BRM, brain metastases only; CSS, cancer-specific survival; LIM, liver metastases only; MOM, multiple organ metastases; OS, overall survival; OTM, other metastases.

In the subgroup analysis, Kaplan–Meier probability plots displayed differences in OS and CSS improvement after immunotherapy in both cohorts and among the five metastatic subgroups in both cohorts (**Figure S1**). As shown in **Figure 3**, immunotherapy had similar effects on the different metastatic subgroups. No improvement in OS was found in the BOM (SEER: Hazard ratio 1.171, 95% CI 0.732–1.874; P=0.510; China: Hazard ratio 1.136, 95% CI 0.637–2.025; P=0.666), LIM (SEER: Hazard ratio 0.737, 95% CI 0.361–1.503; P=0.401; China: Hazard ratio 0.779, 95% CI 0.312–1.946; P=0.594), or OTM (SEER: Hazard ratio 0.923, 95% CI 0.606–1.408; P=0.712; China: Hazard ratio 0.730, 95% CI 0.444–1.200; P=0.214) subgroups. However, immunotherapy significantly increased OS in the BRM (SEER: Hazard ratio 0.565, 95% CI 0.385–0.829; P=0.004; China: Hazard ratio 0.536, 95% CI 0.312–0.920; P=0.024) and MOM (SEER: Hazard ratio 0.546, 95% CI 0.308–0.968; P=0.038; China: Hazard ratio 0.467, 95% CI 0.238–0.914; P=0.026) subgroups. Immunotherapy significantly improved CSS in the BRM (SEER: Hazard ratio 0.551, 95% CI 0.370–0.820; P=0.003; China: Hazard ratio 0.540, 95% CI 0.307–0.950; P=0.033) and MOM (SEER: Hazard ratio 0.524, 95% CI 0.290–0.947; P=0.032; China: Hazard ratio 0.469, 95% CI 0.235–0.937; P=0.032) subgroups, but not in the BOM (SEER: P=0.334; China: P=0.441), LIM (SEER: P=0.301; China: P=0.357), or OTM (SEER: P=0.868; China: P=0.489) subgroups (**Figure 3**).

We also merged the data from the two cohorts. Adjuvant immunotherapy improved OS (Hazard ratio 0.729, 95% CI 0.620–0.856; P<0.001) and CSS (Hazard ratio 0.726, 95%CI 0.612–0.862; P<0.001) significantly in the merged cohort (**Figure 3**). We found different benefits from immunotherapy within each metastatic subgroup. This benefit was greatest in the BRM (OS: Hazard ratio 0.565, 95% CI 0.385–0.829; P=0.004; CSS: Hazard ratio 0.536, 95% CI 0.312–0.920; P=0.024) and MOM (OS: Hazard ratio 0.565, 95% CI 0.385–0.829; P=0.004; CSS: Hazard ratio 0.536, 95% CI 0.312–0.920; P=0.024) subgroups. In the BOM, LIM, and OTM subgroups, immunotherapy did not result in significant differences in OS or CSS.

## Discussion

We analyzed the prognostic significance of immunotherapy at different sites in patients with oligometastatic NSCLC from the SEER and the single-center cohorts. To our knowledge, this was the largest sample size, and the first analysis to assess the role of immunotherapy in treating oligometastatic NSCLC according to different metastatic sites. Immunotherapy is US Food and Drug Administration approved for use alone or in combination with chemotherapy for patients with NSCLC, and has been evaluated after locally ablative therapy for oligometastatic disease (12). However, phase III clinical studies are still lacking in the field of immunotherapy treatment of oligometastatic NSCLC. Whether immunotherapy can confer a survival benefit in patients with oligometastatic NSCLC remains controversial, particular in those with different metastatic sites. Our study showed that immunotherapy improved OS and CSS in patients with oligometastatic NSCLC. These findings agreed with previous findings of improved survival following treatment of metastatic NSCLC with PD-1 inhibitors (15, 16). Recent clinical trials focused on use of immunotherapy to treat various solid tumors have shown a clear association between immunotherapy and increased OS and improved prognosis (17, 18). We also observed reduced OS in patients in the LIM and MOM subgroups. This was consistent with the findings of previous studies showing that patients with NSCLC that had metastasized to multiple organs or the liver had worse OS relative to those with other metastases (19–22). These results showed the value of immunotherapy for treatment of oligometastatic NSCLC patients with liver and multiple organ metastases.

We found that being male was significantly associated with poorer prognosis, which agreed with the results of a study by Radkiewicz C et al, who reported that men with NSCLC had consistently poorer lung cancer-specific survival across stages (23). Moreover, our results showed that patient prognosis was related to tumor location, and patients with tumors located in the main bronchus had worse outcomes than those who had tumors in other locations, which agreed with the results of a study by Li C et al (24). A recent study showed that chemotherapy combined with immunotherapy had superior efficacy for improving anticancer activity (25). Radiotherapy is not an independent prognostic factor for improvement of OS and CSS, due to the remarkable effects of recently developed targeted therapies.

The definition of oligometastatic NSCLC differed across many previous studies, with the maximum number of metastases ranging from 3 to 6 (26). Recently, a number of studies have shown that LT improved the prognosis of patients with oligometastatic NSCLC (27, 28). However, several studies have also shown that immunotherapy was effective for treatment of patients with oligometastatic NSCLC, especially for those at high risk of distant metastases (29, 30). In the most recent NCCN guidelines, immunotherapy is a recognized front-line treatment approach for metastatic NSCLC (31). Therefore, we studied the efficacy of immunotherapy for treatment of oligometastatic NSCLC based on different metastatic sites.

Previous studies focused on metastatic NSCLC have demonstrated that different sites of metastasis can predict clinical outcomes (20, 21, 32).Patients with NSCLC with bone metastasis have the best prognosis (32), which we further confirmed in the present study. We found that patients in the LOM and MOM subgroups had the worst OS and CSS compared to those in other subgroups, which was consistent with the results of a study by Yang J et al, which identified that the mortality risk was highest with MOM and liver metastases (20). We also found that immunotherapy improved OS and CSS in the BRM and MOM subgroups. A recent study found that local control and overall survival were both improved in patients with NSCLC who received concurrent immune checkpoint inhibitor treatment with radiosurgery (33), which was consistent with our results. Another study (34) that brain irradiation induces the strongest immune activation effects and patient outcome compared with those achieved through irradiation of other organs, which may be consistent with our results. However, patients in the LOM subgroup did not benefit from immunotherapy, which may have been associated with poorer response of liver metastasis to immunotherapy. A study reported that liver metastases diminish immunotherapy efficacy systemically in patients and preclinical models, resulting in a systemic immune desert through siphoning of activated CD8+ T cells from the systemic circulation (35). Our results showed that patients in the MOM subgroup benefited most from immunotherapy, which was consistent with the results of a study by Ma SC et al (36), who reported that increased number of metastatic sites (≥2) we associated with pronounced OS benefits from atezolizumab versus docetaxel. This may indicate that these patients represent a subgroup that may benefit from aggressive treatment.

Our study had several limitations. First, the SEER database does not provide a detailed description of the specific immunotherapy used. Second, limited information is provided by the SEER database, and there may be some degree of error in the coding of individual patients. Transfer of patients in a particular group (bone, brain, liver) were restricted to this organ, although patients may have had metastases at other sites not explicitly documented in the SEER database (the most common unrecorded site may be the adrenal). Finally, this study was a retrospective study, and the efficacy of immunotherapy in oligometastatic NSCLS with different metastatic sites should be evaluated in prospective clinical trials.

## Conclusion

Our study showed that immunotherapy improved OS and CSS in patients in the SEER and the single-center cohorts. Furthermore, we found that immunotherapy improved the prognosis of patients in the BRM and MOM subgroups, but not those in the BOM, OTM, or LIM subgroups. The MOM subgroup experienced the greatest benefit from immunotherapy in both cohorts. Therefore, metastatic sites may affect the efficacy of immunotherapy. These subgroups may benefit from aggressive treatment, and our results may have implications for clinical guidance. However, our findings need to be validated in future large-sample prospective clinical trials.

## Data availability statement

The original contributions presented in the study are included in the article/**Supplementary Material**. Further inquiries can be directed to the corresponding author.

## Ethics statement

Ethical review and approval was not required for the study on human participants in accordance with the local legislation and institutional requirements. Written informed consent for participation was not required for this study in accordance with the national legislation and the institutional requirements. Written informed consent was not obtained from the individual(s) for the publication of any potentially identifiable images or data included in this article.

## Author contributions

J-CM, J-XZ, FW, DC and JY contributed to the study. DC and JY designed the project and approved the final manuscript. J-XZ and FW are responsible for modifying and editing this article. J-CM collected the clinical patients information and drafted the article. All authors contributed to the article and approved the submitted version.

## Funding

This work was supported by National Natural Science Foundation of China (82172676), Science Foundation of Shandong (ZR2021YQ52, ZR2020LZL016), Foundation of Bethune Charitable Foundation (2021434953), and the Young Elite Scientist Sponsorship Program by CAST (YESS20210137).

## Conflict of interest

The authors declare that the research was conducted in the absence of any commercial or financial relationships that could be construed as a potential conflict of interest.

## Publisher’s note

All claims expressed in this article are solely those of the authors and do not necessarily represent those of their affiliated organizations, or those of the publisher, the editors and the reviewers. Any product that may be evaluated in this article, or claim that may be made by its manufacturer, is not guaranteed or endorsed by the publisher.

## Supplementary material

Supplementary Figure 1 Kaplan-Meier probability plots of Overall Survival and Cancer Specific Survival between the Immunotherapy group and the No immunotherapy group within different metastatic site subgroups: BOM group (SEER: a and b; Single-Center: k and l), BRM group (SEER: c and d; Single-Center: m and n), LIM group (SEER: e and f; Single-Center: o and p), MOM group (SEER: g and h; Single-Center: q and r), and OTM group (SEER: i and j; Single-Center: s and t). *Abbreviations*: MOM, multiple organ metastases; OTM, other metastases.
